# Feasibility of Virtual Reality based Training for Optimising COVID-19 Case Handling in Uganda

**DOI:** 10.21203/rs.3.rs-882147/v1

**Published:** 2021-10-04

**Authors:** Paul Buyego, Elizabeth Katwesigye, Grace Kebirungi, Mike Nsubuga, Shirley Nakyejwe, Phillip Cruz, Meghan McCarthy, Darrell Hurt, Andrew Kambugu, Joseph Walter Arinaitwe, Umaru Ssekabira, Daudi Jjingo

**Affiliations:** Infectious Disease Institute, Makerere University; African Center of Excellence in Bioinformatics and Data Intensive Sciences, Makerere University, Uganda; National Institute of Allergy and Infectious Diseases; Infectious Disease Institute, Makerere University; African Center of Excellence in Bioinformatics and Data Intensive Sciences, Makerere University, Uganda

**Keywords:** Virtual Reality, COVID-19, Personal Protective Equipment, Medical Education, Pandemics

## Abstract

**Background:**

Epidemics and pandemics are causing high morbidity and mortality on a still-evolving scale exemplified by the COVID-19 pandemic. Infection prevention and control (IPC) training for frontline health workers is thus essential. However, classroom or hospital ward based training portends an infection risk due to the in-person interaction of participants. We explored the use of Virtual Reality (VR) simulations for frontline health worker training since it trains participants without exposing them to infections that would arise from in-person training. It does away with the requirement for expensive Personal Protective Equipment (PPE) that has been in acute shortage and improves learning, retention and recall. This represents the first attempt in deploying VR-based pedagogy in a Ugandan medical education context.

**Methods:**

We used animated VR-based simulations of bedside and ward-based training scenarios for frontline health workers. The training covered the wearing and stripping of PPE, case management of COVID-19 infected individuals and hand hygiene. It used VR headsets and Graphics Processing Units (GPUs) to actualize an immersive experience, via a hybrid of VR renditions and 360degrees videos. We then compared the level of knowledge acquisition between individuals trained using this method to comparable cohorts previously trained in a classroom setting. That evaluation was supplemented by a qualitative assessment based on feedback from participants about their experience.

**Results:**

The effort resulted into a well-designed COVID-19 IPC VR curriculum, equivalent VR content and a pioneer cohort of trained frontline health workers. The formalized comparison with classroom-trained cohorts showed relatively better outcomes by way of skills acquired, speed of learning and rates of information retention (*P-value =4.0e-09*) - suggesting the effectiveness and feasibility of VR as a medium of medical training. Additionally, in the qualitative assessment 90% of the participants rated the method as very good, 58.1% strongly agreed that the activities met the course objectives, and 97.7 % strongly indicated willingness to refer the course to colleagues.

**Conclusion:**

VR-based COVID-19 IPC training is feasible, effective and achieves enhanced learning while protecting participants from infections within a pandemic context in Uganda. It is a delivery medium transferable to the contexts of other highly infectious diseases.

## Background

In the first two decades of the 21^st^ century, humanity has witnessed disease outbreaks that have highlighted the danger of chronic vulnerability to infectious diseases, known and unknown. These epidemics and pandemics have resulted in high morbidity and mortality on a still-evolving scale[1]. Among these are SARS CoV, MERS, Ebola, Marburg, cholera, influenza, and current SARS CoV-2[1]. Uganda is particularly prone to infectious disease outbreaks[2, 3]. Between 2000 and 2016, Uganda reported eight outbreaks caused by filoviruses (Ebola Virus Disease(EVD) and Marburg), more than any other country in the world[4]. Other outbreaks experienced between 2017 and 2019 included yellow fever, anthrax, rift valley fever, meningitis, avian influenza, crimean-congo hemorrhagic fever (CCHF) and EVD[4]. In December 2019, a novel coronavirus (SARS-CoV-2, the causative agent for COVID-19 emerged in China and became a pandemic with the first case in Uganda reported in March 2020[5, 6].

Healthcare workers (HCWs), support staff, patients, and visitors to health facilities are at risk of acquiring such infections in healthcare settings (out-patient and in-patient departments, HIV care clinics, and operating theatres) as well as from the community[3]. With the current COVID-19 pandemic, 2299 health workers so far have contracted the disease, and 28 have died, reported as of 14^th^ June 2021[7]. To minimize the risk of transmitting these infectious agents from one person to another, health workers to patients and vice versa, infection prevention and control (IPC) practices should be paramount[3]. IPC is a practical, evidence-based approach that focuses on preventing patients, visitors, and HCWs from being harmed by avoidable and preventable infections in a healthcare setting[3].

IPC practice in Uganda’s health facilities is still limited, demonstrated by widespread noncompliance to hand hygiene measures, poor waste management, lack of isolation protocols, and lack of functional IPC committees[2, 8, 9]. For example, at 20–50%, hand hygiene (HH) compliance is still much lower than the international standard of 80%, as demonstrated by IPC assessments at different health facility levels[2, 9–11]. Although there was near universal improvement in HH compliance between 2018 and 2019 (Table 1), the improvement scores were still significantly lower than the standard of 80%. Observed improvements were partly due to improved availability of alcohol based hand rub in some hospitals and capacity building activities that targeted the hospitals’ IPC committees[12].

In trying to bridge this gap, Uganda developed an Ebola Contingency Plan and implemented heightened highly infectious diseases preparedness activities to enhance operational readiness to handle any highly infectious cases. The Ministry of Health (MoH) mobilized resources for the response and coordinated stakeholders to: perform classroom training of teams on surveillance case management and set up nine national highly infectious diseases treatment units. Between 2018 and 2019 MoH trained 526 health workers in EVD case management; mentored 9,806 health workers in 562 health facilities across 11 districts, and 18 safe and dignified burial teams were constituted and trained[13]. It also trained 716 health workers in EVD surveillance, and 719 contact tracers in 26 districts. Nineteen laboratory technicians from Uganda National Health Laboratories Services (UNHLS), Uganda Virus Research Institute (UVRI), and National Tuberculosis Reference Lab trained in Ebola diagnostics – RDTs and gene expert. 7,575 Village Health Trainers(VHTs) trained in Community Based Disease Surveillance (CBDS) in six districts[13]. However, an EVD (representing highly infectious diseases) functional simulation exercise conducted in April 2019 to assess readiness capacities built found that there were still glaring gaps in the preparedness efforts[13]. It established weak infection prevention and control practices in districts where trainings had been conducted. Recommendations from the simulation exercise point towards further efforts in highly infectious diseases preparedness activities to strengthen and improve the country’s readiness [13].

The classroom-training model used was hectic, takes a longer time and often does not provide the actual rendition of a highly infectious setting. As a result, the health workers do not only take a longer time to learn the skills, they are also less likely to retain those skills. We therefore proposed and pioneered the use of virtual reality (VR) simulations for frontline health worker training in Uganda.

VR is the use of computer technology to create a simulated environment that immerses the user into an experience. It uses dynamic 3D-visualization to actualize a near real-world rendition of the circumstances and context[14, 15]. The technology can be used to create a near-real world environment or even simulations difficult or expensive to actualize in conventional physical reality. VR allows for the use of multiple senses (e.g., touch, hearing, seeing and sometimes smell), which are used simultaneously during the learning process[16, 17]. This could improve the engagement and mental alertness of both the students and teachers based on the significant interaction effect between the learning mode and the learners[18]. Consequently, it speeds up the rates at which individuals assimilate information and increases the extent of information retention[19, 20]. That information assimilation is driven by the technology’s ability to; more precisely simulate features and processes, give learners real-time interactive feedback and give extreme close-up and dynamic multi-perspective views of objects. Accordingly, VR is broadly applicable and has been applied to many different areas of education including technology training[21], natural sciences, history and architecture[21]. It has been described as the learning aid of the 21st century[22]. Perhaps medicine represents one of the fields where VR has proven most effective owing to that field’s dependence on elaborate illustrations of anatomic and physiological features[23, 24]. Indeed, studies done on the use of VR in medical education have shown it to yield favorably comparable outcomes in terms of knowledge and skills gain in comparison to classroom instruction[25]. Furthermore, additional studies have demonstrated that VR improves post intervention knowledge and skills outcomes of health professionals when compared with traditional education or other types of digital education[25]. As a result, VR is increasingly being adopted as a supplementary medium for medical training in Europe and North America [22, 26]. Forexample, the increasing financial feasibility of VR has allowed for educational institutions to incorporate the technology into their training at 96% of the universities in the UK[27] and it was forecast to reach over 95 million users in the US by 2022 [28].

However, the feasibility of VR in low-resourced, less endowed biomedical/health education systems with lower technology exposure and technology culture like Uganda has not been tested partly because of a prior of lack of requisite equipment and VR skilled individuals to develop and manage its platforms. The recent emergence of multiple epidemics in several low resourced environments including Uganda has added to the urgency to test its feasibility in such environments due to its ability to enable effective training of health workers with a minimum risk of infection. In this paper, we demonstrate the feasibility of such an approach for training health care workers in IPC within the context of an active highly infectious COVID-19 pandemic in a resource limited setting. We do so by conversion of some modules of COVID-19 IPC classroom curriculum into VR mode and piloting them with a cohort of pioneer participants using an improvised hybrid of VR and 360° videos. The cohort is then used to assess both the feasibility and effectiveness of VR in such settings.

## Methods

### General approach

The work was implemented through VR based simulation of animated IPC bedside and ward training scenarios for critical health workers. The training covered the handling of VR equipment, wearing and stripping of Personal Protective Equipment (PPE), case management of individuals infected with COVID-19 and hand hygiene, all while ensuring maximum safety from infection and improving access to the training. It used VR headsets with head-mounted displays, connected to VR software into which content was programmed to provide a fully immersive experience that was piloted on a pioneer cohort of participants.

### Curriculum development and platform preparation

The COVID-19 IPC Virtual Reality course was developed using the updated Ministry of Health COVID-19 IPC classroom/in-person course as the guide. Essential topics and skills were extracted and designed into a customized preliminary curriculum map with particular attention to skills amenable to VR renditions. The developed curriculum map was then strengthened with additional feasible simulations and renditions, such as that of the COVID-19 virus particle and its anatomy. The resultant curriculum map covered five modules; Introduction to VR equipment, Introduction to the SARS-Cov-2 virus, Infection Prevention and Control, Disinfection and waste management, and COVID-19 case management (Table 2). The updated IPC training materials corresponding to each curriculum module were then transformed into VR space as artifacts through an artifact design, coding and launching step. Each artifact was supplemented with requisite activities and narratives which were synchronously added to the dynamic artifacts and then voiced over. The aggregation and alignment of those three information channels: the artifacts, activities and audio narratives were enabled by the relevant *Enduvo* VR software platform functionalities and consequently embedded into the Enduvo VR platform as virtual reality modules.

### Pre-piloting

Using the developed VR content, a pre-pilot training of the Infectious Disease Institute (IDI) and MoH IPC expert staff was conducted to gain prior feedback and assessment of the course. This enabled fine tuning the course before it would be rolled out to the field clinicians. It was conducted on 10 participants over a 1week period. Insights gained from this exercise included; the need to continuously review and update content to align with current best COVID-19 IPC practices since WHO and CDC kept updating recommended preventive measures over the course of the pandemic, integrate safety measures during use of the shared VR equipment and improve quality of the 360° videos. To take care of these shortcomings, we revised and updated the content, procured fluid resistant head nets, eye masks and alcohol hand rub for each VR station. We also re-made some of the 360° videos to improve quality.

### Piloting

**Figure 1:**
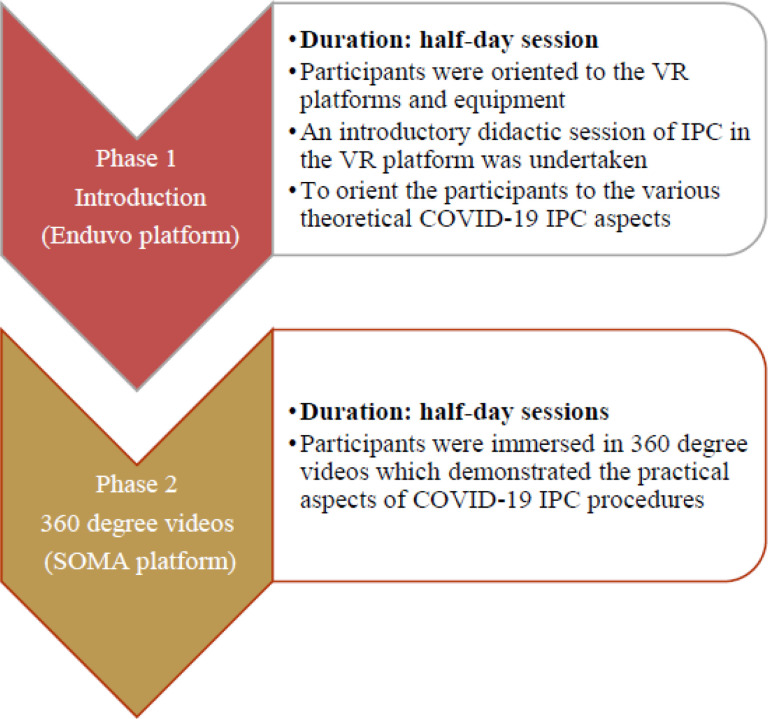
Phase one and two of the pilot training

Having integrated solutions to shortcomings raised at the pre-pilot level, the exercise was then rolled out to field clinicians as the substantive pilot training. Field clinicians constituted a multidisciplinary team including medical officers, nurses, laboratory, clinical officers, pharmacists and epidemiologists. The pilot training of field clinicians was conducted in two phases for a period of two weeks with a ratio of 1 VR instructor to two participants a day. Phase one consisted COVID-19 IPC course theory in the immersive Enduvo VR platform for a duration of 1 week and Phase two comprised practical training using 360° videos to complement phase one - for a period of one week. The successful implementation of the second phases (Fig. 1) set the stage for a more fully-immersive VR third phase.

#### Phase 1:

This was the introductory phase of this course. It served to orient participants to the VR platforms and equipment and used a didactic approach to cover the theoretical principles of COVID-19 IPC. Each group of participants received a demonstration of the process of navigation of the Enduvo VR platform showing how to load sessions, navigate between sessions, take a break, do assignments and submit assignments. The phase was delivered by course instructors, who provided both instructional and troubleshooting support to the participants in cases of device or procedural glitches during the course.

Once the participants were comfortable with navigation and manipulation of the platforms, they covered the didactic introductory sessions in the VR platform to orient them to the various theoretical underpinnings of COVID-19 IPC. These were eleven short, objective guided topics, each with five minutes of run time. However, the actual topic duration was variable as it was self-paced with individual participants having room to pause, forward, rewind and navigate the embedded VR artifacts (Table 3) and videos for their own clarity. At the end of each objective session an assessment in the form of a knowledge based multiple choice question was undertaken to assess knowledge acquisition. This phase took half a day for each trainee.

#### Phase 2:

During this phase, participants covered the practical aspects of COVID-19 IPC and case management. This was done through demonstrations of the procedures using 360° videos in combination with a full immersion VR prototype for hands on experience. The 360° videos helped participants to view pre-recorded IPC and case management scenarios in an enhanced 3D experience which affords them a better and near-real engrossment with the scenario.

This phase involved health workers exploring the 360° VR videos several times in the VR lab until a required level of confidence in performing the task was achieved. At the end of each session an objective assessment in the form of a knowledge based multiple choice question was undertaken to assess knowledge acquisition. Finally, participants were given a demonstration of a full immersion practical VR prototype that guided them through actual practice of IPC activities. This was implemented through a collaboratively developed VR prototype in which each IPC procedure is repeated until the desired level of competence is achieved. It included an immersive, dynamic and interactive VR environment which flags participants every time they get a step wrong through an audio booze and doesn’t let them proceed until they have perfected the procedure. This phase took half a day for each trainee. The lab is actively procuring equipment to support the full conveyance of this phase.

### Training structure

The phases were delivered as group sessions consisting of a maximum of six participants each to meet social distancing standards. There were 10 such groups of six participants, with two groups attending the course each day. Each cohort attended phase 1 for half a day in the first week and phase 2 for half a day in the second week. All the sessions were self-paced and each trainee had the opportunity to iteratively go through each session until they had grasped the content. A VR technical assistant was present to guide the participants throughout their sessions.

### Class size

We targeted to train 60 frontline health care workers practicing in the IDI supported health care facilities within Kampala and Wakiso districts. The sample size and cohorting was determined by the six available active number of training stations within the laboratory, all of which are over four meters from each other. As each group of six participants needed half-day for training, the laboratory could only handle 12 participants a day. Accordingly, in 5 days of the work-week, the lab could handle a maximum of 60 participants with appropriate social distancing. Coupling the content with 3D web technology and internet connectivity would allow for scaling the training by orders of magnitude, especially in cases where prospective participants have appropriate devices like smart phones to receive 3D web content.

### Course evaluation

This VR training was evaluated using three broad approaches. In the first approach, learning outcomes in terms of knowledge acquisition and knowledge retention by participants was assessed by comparing similar outcomes in previous cohorts that were trained using the classroom instructional model. The second approach was an experiential assessment designed to gauge the experience of the participants, particularly because this is a new pedagogical approach and technology that many were experiencing for the first time. In this assessment, an online individual survey form was used to evaluate the course. The objective of this survey was to obtain participants’ feedback on the overall implementation of the course including strengths, weaknesses and recommendations for the various components to inform improvements to subsequent courses. In the survey, we used a Likert scale questionnaire with – number of choices ranging from strongly agree to strongly disagree. The survey consisted of a set of eight multiple-choice questions and four open-ended questions that gave participants an opportunity to express their views in detail.

## Results

The exercise resulted into a VR-based IPC curriculum map (Table 2), corresponding VR and 360° content, 6 practiced VR instructors, corresponding VR and 360° content and a pioneer cohort of 52 well trained frontline health workers.

### Trained cohort diversity

**Figure 2:**
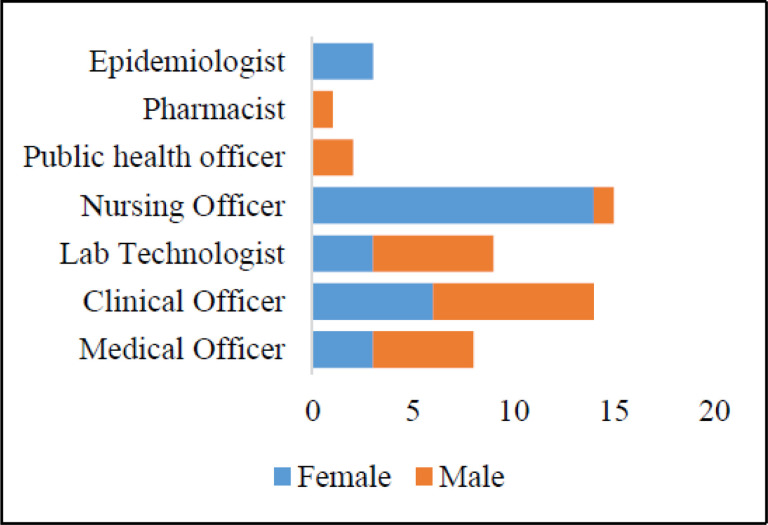
Gender and vocational attributes of participants

52 frontline health workers participated and completed, with 27 (60%) from government institutions, 23(44%) from a non-government organization (NGO) and 2 (3.6%) from private facilities. The majority of participants were females at 31(56.4%) and males were 24 (43.6%) (Fig. 2). The vocation representation included nurses at 15 (28.9%), followed by clinical officers at 14 (26.9%) laboratory officers 9 (17.3%), Medical Officers 8 (15.4%), Public Health Officers 2 (3.9%), Pharmacists 1 (1.9%), and Epidemiologists 3 (5.8%) (Table 4). The average age was 34.6 with the youngest at 25 and oldest at 60, and a median age of 31. Taken together, the participants constituted a professionally diverse cohort with regard to gender, age, type of institution of employment and vocation.

### Knowledge and skills acquisition are feasible in VR based training

**Figure 3:**
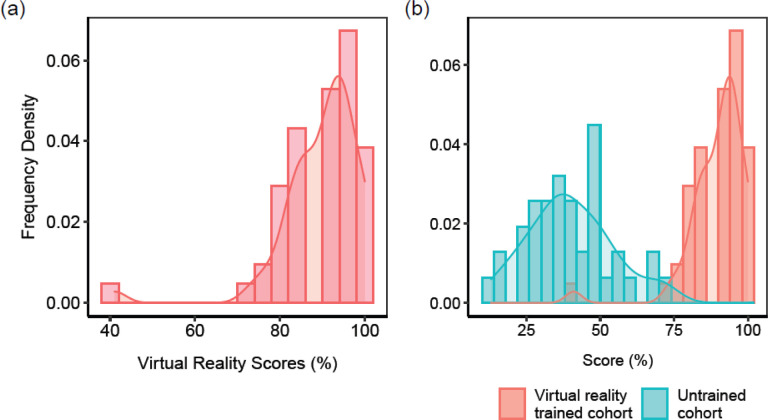
Score comparisons (a) Distribution of scores for 52 individuals after undergoing VR training (b) A comparison of the bottom baseline of an untrained cohort (blue bars) with a comparable VR-trained cohort

The completion rate of the course was 98.1% (52 participants). The highest score was 100% in both phases with the lowest being 41% in phase 1 and 33% in phase 2. The average score was 90% and 79.9% in phase 1 and phase 2 respectively. These relatively high scores, where the overwhelming majority of participants scored above 80% (Fig. 3a) suggest the effectiveness and feasibility of VR as a medium of medical training in this low resourced setting. Indeed, when checked against the bottom baseline (a comparable cohort of untrained individuals), there is a clear and significant improvement by way of skills and knowledge gained (Fig. 3b) *p-value =* ***4.0E-35***.

### Qualitative experiential assessment

**Figure 4:**
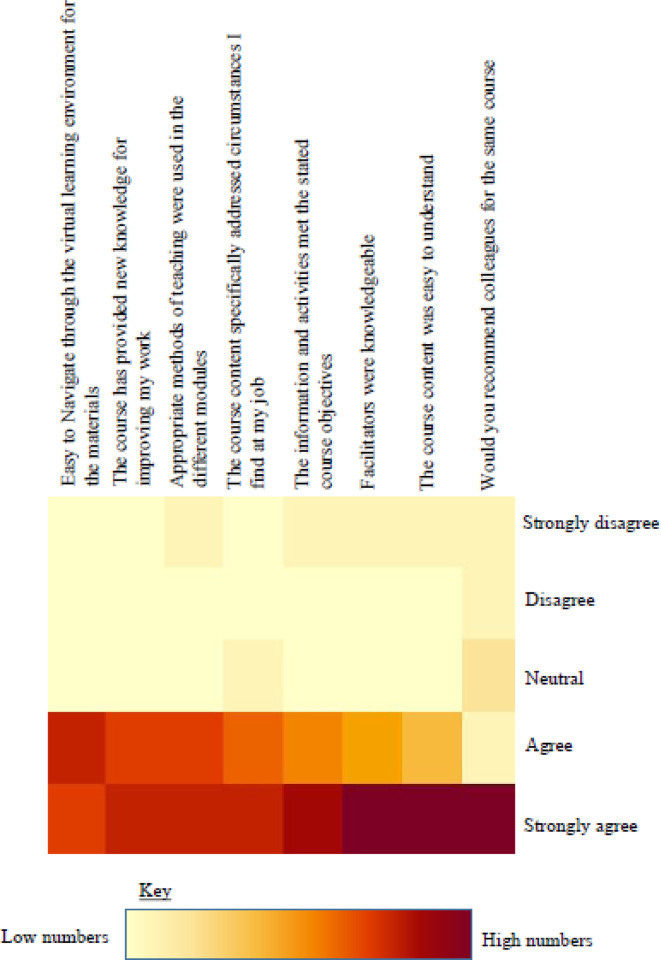
Qualitative assessment of VR-based training

Feasibility also needed to be assessed in terms of the levels of comfort with the technology by the participants, which impacts their ability to learn through it as well as its likelihood for acceptance as a training platform by the wider community of health workers. This assessment was done through seeking qualitative feedback from the participants. We received feedback from 80% of the participants, covering whether they felt it met their course expectations, whether it was navigable, whether they felt comfortable learning from it and if they would recommend it to colleagues (Fig. 4). Overall, 58.1% strongly agreed that the information and activities met the course objectives and 65.1 % strongly agreed that the course content was easy to understand. Over 90% agreed that the methods for training used were very good and 46.5% of those strongly agreed that appropriate methods were used for the pilot. 46.5% strongly agreed that it was easy to navigate the VR environment for the materials and 97.7 % of the participants strongly indicated willingness to refer the course to a colleague (Fig 4). As might be expected, the younger participants and those with preexisting expertise in other related technology found it easier to navigate the platform and hence complete the course faster. Similarly, participants with visual challenges expressed extra concerns on visibility of the artifacts. In the open-ended questions, participants echoed that the VR mode of learning was less hectic, because there was no need to write, they had control of their learning in a single space, there was better concentration in the virtual environment with no interruptions, and it was more practical while utilizing few resources like PPE and stationary. Taken together, the results of these qualitative assessments reveal a net positive experience of the platform and suggests a significant likelihood for its acceptance as a channel of learning among frontline health care workers in Uganda.

### VR-based training is a comparatively competitive medium of training

**Figure 5:**
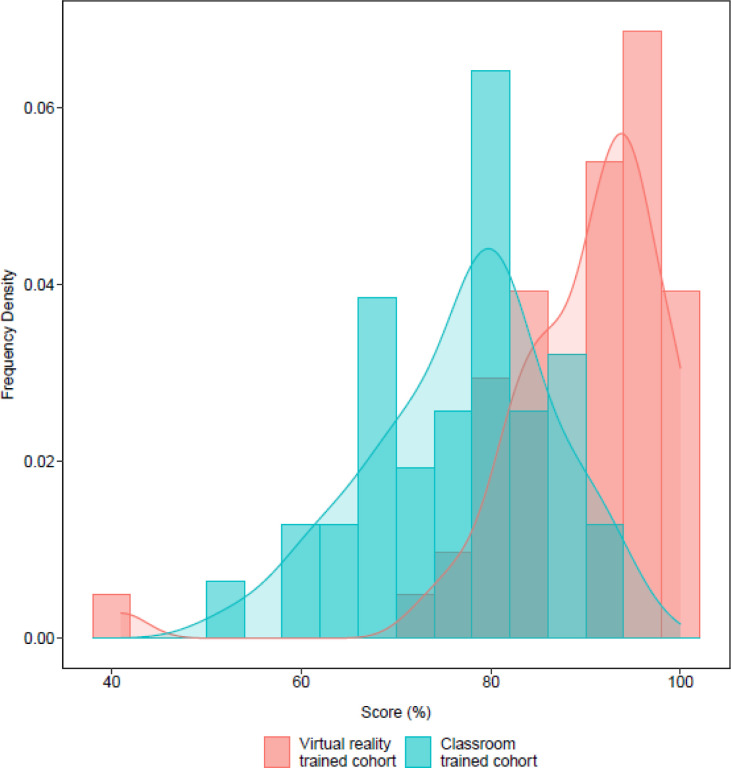
Comparison of the classroom-trained cohort (blue bars) with the VR-trained cohort (red bars)

Having established the viability of VR training for frontline health workers in the context of COVID-19 in a low-resourced environment, we sought to establish how it compares as a pedagogical medium with the pre-existing method of classroom based instruction. To do that, we drew a similar cohort from health workers we had previously trained using classroom instruction. The cohort was comparable to the VR-trained individuals in terms of the number of participants, their gender distribution, age distribution and vocational background as well as topics covered during the training. As expected, the distribution of scores for both classroom instruction and VR trained cohorts was higher than the bottom baseline of the untrained cohort (Fig. 3b). However, despite a partial overlap, the scores for the VR-trained cohort were statistically significantly higher than those of the classroom based cohort (Fig. 5) *(p-value =* ***4.0E-09****)*. That difference in learning outcomes suggests VR training to have a comparative advantage relative to classroom instruction for COVID-19 IPC procedures among frontline health workers in Uganda. This result is also in line with previous findings showing VR to be a favorably comparable training medium[19, 25].

## Discussion

One of the major motivations of this study was to determine the feasibility of VR training in resource-constrained environments. This training piloted virtual reality technology as a new pedagogical approach that presents an alternative training medium for improving competence and optimizing safety of COVID-19 handling and case management in Uganda. The high scores on the post training test for knowledge and skills acquisition attest to the effectiveness and feasibility of the technology for medical training in a low resource setting. This is in agreement with studies in highly resourced settings that have shown that knowledge gain and skills acquisition using VR training approach was non inferior to traditional classroom training model [27].

Constraints to VR training in this low-resourced environment included limitations in terms of technology, a paucity of skilled instructors and the lack of a trainee cohort with prior exposure to VR technology. With the improvisations of supplementing VR with 360° videos, we developed a hybrid platform that enabled training even without the full VR complement owing to absence of the latest VR headsets with optimal functionality and the limited VR programming capacity. Furthermore, time was invested in training a critical mass of instructors to learn the use of the gadgets and their appropriate and optimal deployment in training sessions. That helped mitigate the constraint of a paucity of well qualified instructors. Equally, to accommodate the fact that participants had no prior exposure to VR, the curriculum was designed to begin with a module that brings the participants up-to speed with the use of VR gadgets. Finally, the modules were also paced and timed to fit within available training time while also maintaining quality of learning.

As the VR technology matures by way of gadgets and programming platforms, and becomes cheaper, the existing shortfalls will continue to fizzle out. This will increase its potential as a training medium via enabling an even stronger 3rd phase (more interactive training). Additionally, its packaging in short modules of 5–10 minutes and its being amenable to self-pacing should improve learning outcomes while reducing training time. Furthermore, its better learning outcomes in comparison to classroom instruction that we demonstrated in the results section has been seen in other contexts that found it either equivalent[25] or superior[19]. Its outstanding scaling challenge in low resourced settings could be mitigated with improvised card-board headsets[29] and translation of content into 3D web space for online transmission to wider pools of participants.

## Conclusion

Taken together, the VR training designs and improvisations applied here, and the pilot results attained, argue for VR as a feasible training medium for medical education in Uganda that is also extendable to other applications as was previously posited[30]. In due course, it is likely to become a mainstay of endeavors such as training, diagnosis, illustration, and enhanced communication.

## Supplementary Material

Supplement 1

## Figures and Tables

**Table 1:** HH compliance at regional referral hospitals in Uganda

Facility	2018	2019
Mbale	9%	46%
Jinja	20%	52%
Naguru	23%	50%
Kabale	24%	40%
Fort Portal	22%	33%
Lira	22%	31%
Arua	24%	30%
Masaka	7%	12%
Mbarara	19%	24%
Gulu	10%	13%
Hoima	18%	20%
Soroti	19%	9%
Moroto	43%	32%
Mubende	54%	24%
**Overall**	**22%**	**30%**

**Table 2:** VR Curriculum Map

Week 1: VR Platform	
Module and sessions	Training duration
1. **Introduction to VR Equipment**	**Total duration 5 min**
1. **Introduction to Sars-Cov-2**	**Total duration 20 min**
1. Understand what COVID-19 is	5 min
2. Describe the pathophysiology of COVID-19	5 min
3. Understand the clinical presentation of COVID-19	5 min
4. Understand screening and triaging of COVID-19 patients	5 min
1. **Infection Prevention and Control**	**Total duration 20 min**
1. Understand what IPC is	5 min
2. Understand the importance of hand hygiene in IPC	5 min
3. Understand respiratory hygiene	5 min
4. Describe Personal Protective Equipment	5 min
1. **Disinfection and Waste management**	**Total duration 10 min**
1. Understand the three levels of decontamination in COVID-19 prevention	5 min
2. Understand the waste management process in a COVID-19 situation	5 min
1. **Understand COVID-19 case management**	**Total duration 5min**
1. Patient management principles	5 min
	
Week 2: 360° videos on SOMA* platform	
Module and sessions	Training duration
**1. Performing hand hygiene**	**Total duration 10 min**
1.1 Alcohol based hand rub	5 min
1.2 Soap and water	5 min
**2. Personal protective equipment**	**Total duration 30 min**
2.1 Gloving and de-gloving	5 min
2.2 Respiratory hygiene (demonstrating mask use)	5 min
2.3 Donning coverall	5 min
2.4 Donning gowning	5 min
2.5 Doffing of coverall	5 min
2.6 Doffing of gown	5 min
**3. Case Management**	**Total duration 5 min**
3.1 Management of Confirmed COVID-19 case at ETU or level II isolation unit	5 min

* SOMA is a platform to host 360 degree videos that we developed locally

## Abbreviations

CBDS: Community Based Disease surveillance
CCHF: Crimean Congo Hemorrhagic Fever
EVD: Ebola Virus Disease
GPU: Graphics Processing Unit
HCWs: Health Care Workers
HH: Hand Hygiene
IPC: Infection prevention and Control
MoH: Ministry of Health
NIH: National Institute for Health
OCICB: Office of Cyber Infrastructure and Computational Biology
PPE: Personal Protective Equipment
RIF: Research Innovations Fund
UK: United Kingdom
UNHLS: Uganda National Health Laboratories Services
USA: United States of America
UVRI: Uganda Virus Research Institute
VR: Virtual Reality
