# Supplementary material for: Feasibility of Virtual Reality based Training for Optimising COVID-19 Case Handling in Uganda
Source: Res Sq. 2021 Oct 4:rs.3.rs-882147. Preprint. [Version 1] doi: 10.21203/rs.3.rs-882147/v1 (PMC8491848; doi:10.21203/rs.3.rs-882147/v1)
Supplement: Supplement 1 [file a9ce6b3d6fe1513240bb516a.docx]

**Table 3** 3D VR Artifacts

| **Artifact** | **Description** | **Role** |
| --- | --- | --- |
| 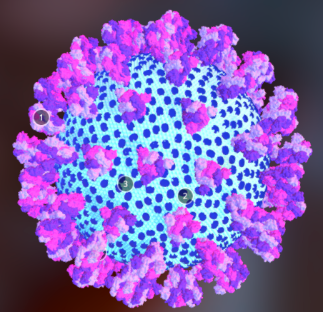 Structure of Sars-COV-2 | 1.Spike protein  2.Membrane protein  3.Envelop protein | Responsible for attachment and entry of the virus into the host cell  promotes completion of viral assembly by stabilizing the N protein-RNA complex, inside the internal virion  plays major role in pathogenesis, virus assembly, and release |
| 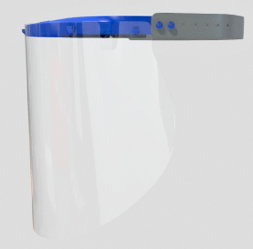  2  1  Face shield | 1.Visor  2. Strap | Protects wearer’s face from contamination  Adjusts the face shield to fit the wearer. |
| 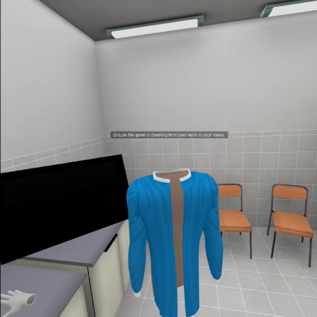 Medical gown | Long sleeved disposable surgical gown with elastic cuffs. | Barrier to fluids and microbial transmission from patient to medical personnel. |
| 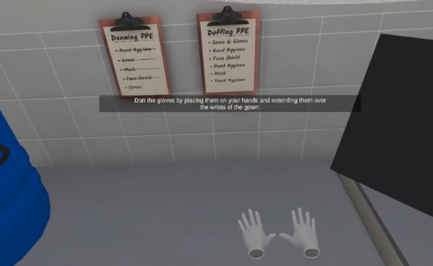 Surgical gloves | Pair of white surgical gloves | protective barrier to prevent the possible transmission of diseases between healthcare professionals and patients during surgical procedures |

**Table 4** Vocational distribution of participants among types of institutions

| Characteristics of participants | n | % |
| --- | --- | --- |
| Occupation (n=52) |  |  |
| Medical Officer  Clinical Officer  Nursing Officer  Public Health Officer  Lab technologist  Pharmacist  Epidemiologist | 8  14  15  2  9  1  3 | 15.38  26.92  28.85  3.85  17.31  1.92  5.77 |

*^government organization^ ^Non-government organization^ ^private organization^*
